# TGF-ß1 Enhances the BMP-2-Induced Chondrogenesis of Bovine Synovial Explants and Arrests Downstream Differentiation at an Early Stage of Hypertrophy

**DOI:** 10.1371/journal.pone.0053086

**Published:** 2013-01-03

**Authors:** Nahoko Shintani, Klaus A. Siebenrock, Ernst B. Hunziker

**Affiliations:** Departments of Orthopaedic Surgery and Clinical Research, Center of Regenerative Medicine for Skeletal Tissues, University of Bern, Bern, Switzerland; University of Texas Southwestern Medical Center, United States of America

## Abstract

**Background:**

Synovial explants furnish an *in-situ* population of mesenchymal stem cells for the repair of articular cartilage. Although bone morphogenetic protein 2 (BMP-2) induces the chondrogenesis of bovine synovial explants, the cartilage formed is neither homogeneously distributed nor of an exclusively hyaline type. Furthermore, the downstream differentiation of chondrocytes proceeds to the stage of terminal hypertrophy, which is inextricably coupled with undesired matrix mineralization. With a view to optimizing BMP-2-induced chondrogenesis, the modulating influences of fibroblast growth factor 2 (FGF-2) and transforming growth factor beta 1 (TGF-ß1) were investigated.

**Methodology/Principal Findings:**

Explants of bovine calf metacarpal synovium were exposed to BMP-2 (200 ng/ml) for 4 (or 6) weeks. FGF-2 (10 ng/ml) or TGF-ß1 (10 ng/ml) was introduced at the onset of incubation and was present either during the first week of culturing alone or throughout its entire course. FGF-2 enhanced the BMP-2-induced increase in metachromatic staining for glycosaminoglycans (GAGs) only when it was present during the first week of culturing alone. TGF-ß1 enhanced not only the BMP-2-induced increase in metachromasia (to a greater degree than FGF-2), but also the biochemically-assayed accumulation of GAGs, when it was present throughout the entire culturing period; in addition, it arrested the downstream differentiation of cells at an early stage of hypertrophy. These findings were corroborated by an analysis of the gene- and protein-expression levels of key cartilaginous markers and by an estimation of individual cell volume.

**Conclusions/Significance:**

TGF-ß1 enhances the BMP-2-induced chondrogenesis of bovine synovial explants, improves the hyaline-like properties of the neocartilage, and arrests the downstream differentiation of cells at an early stage of hypertrophy. With the prospect of engineering a mature, truly articular type of cartilage in the context of clinical repair, our findings will be of importance in fine-tuning the stimulation protocol for the optimal chondrogenic differentiation of synovial explants.

## Introduction

Articular cartilage is characterized by unique architectural and compositional properties which reflect its highly specialized biomechanical functions within synovial joints. These morphological and biochemical peculiarities are, however, achieved at a high cost: adult tissue that has been injured by trauma or disease possesses a very limited capacity for self-repair. In young adults, structural damage to the articular-cartilage layer is a common consequence of sports injuries, occupational accidents and obesity [1]–[3], whereas in elderly individuals, it is one of the early manifestations of degenerative joint diseases, including osteoarthritis [4].

Various tissue-engineering approaches are now being pursued with a view to promoting the repair of articular-cartilage lesions [5], [6]. Owing to their great capacity for self-renewal, strong chondrogenic potential and accessibility, mesenchymal stem cells (MSCs) are an attractive option for the engineering of cartilage tissue in the context of repair [7], [8]. MSCs were originally isolated from bone marrow [9], but they are now known to reside within many different mammalian tissues, such as the synovium, periosteum, trabecular bone, articular cartilage, muscle and fat [10]–[17]. Under appropriate stimulation conditions *in vitro*, MSCs can differentiate into several tissue types of mesenchymal origin, including bone, cartilage and fat.

By virtue of its location within the joint cavity, the synovium would be an ideal source of MSCs for the repair of articular-cartilage lesions. Both the synovium and articular cartilage develop from a common pool of MSCs [18]. Hence, MSCs of synovial origin might give rise to a type of articular cartilage that is more joint-specific than that formed from those of an ontogenically unrelated tissue source. Moreover, given that MSCs originating from the synovium have been shown to have a higher chondrogenic capacity than those derived from either bone marrow, periosteum, fat or muscle [19], and that this tissue can undergo spontaneous repair after its damage or partial removal [20]–[22], strips could be excised and directly autotransplanted to the defect site, thereby obviating the need to isolate the MSCs and to expand them *in vitro*. Furthermore, the physiological scaffold that is afforded by the synovial extracellular matrix might be more conductive to chondrogenic differentiation than an artificial carrier or a natural one that has been derived from a non-joint-associated tissue. In line with this tenet are the observations that the synovium can differentiate into cartilaginous tissue under both clinicopathological [23], [24] and experimental conditions [25]–[27], as well as our own findings: in previous studies, explants of synovial tissue were shown to be capable of forming a more abundant cartilaginous matrix than isolated synovial cells that had been cultured in alginate or as aggregates [27]–[30].

In previous studies of ours [27], [30], the chondrogenic differentiation of MSCs was shown to be influenced by their tissue source (synovium vs. bone marrow), by the absence or the presence of their natural extracellular matrix (aggregates of isolated cells vs. tissue explants), by the nature of the stimulant [bone morphogenetic protein 2 (BMP-2) vs. transforming growth factor beta 1 (TGF-ß1)], and by the inclusion or the exclusion of the modulating agent dexamethasone (DEX). Hence, in order to elaborate a synovial-explant-based system for the repair of cartilage, it will be necessary to delineate an optimal stimulation protocol for the formation of a hyaline type of tissue.

In the aforementioned studies [27], [30], BMP-2 was shown to induce the chondrogenic differentiation of cultured bovine synovial explants only when DEX was excluded from the medium. Since BMP-2 was more potent than TGF-ß1 in inducing not only the expression of the gene for type-II collagen but also the post-translational production and secretion of the protein itself, it would appear to be the more promising candidate of the two for the generation of a hyaline type of cartilage from synovial explants. However, BMP-2 alone was unable to effect the complete differentiation of synovial explants into a typically hyaline type of articular cartilage throughout the entire tissue volume. Moreover, the synovial cells underwent full downstream differentiation into the terminal hypertrophic state, which is tantamount to calcification of the extracellular matrix. Uninterrupted hypertrophy carried to completion is characteristic of growth-plate cartilage [31], [32], in which it is a necessary precondition for mineralization and the longitudinal growth of bones. But in articular cartilage, hypertrophy is arrested at an early stage [33], [34], specifically to avert matrix mineralization. Hence, for the generation of a hyaline type of articular cartilage from synovial explants, the use of BMP-2 as the sole stimulant does not suffice. We hypothesize that the simultaneous application of an additional – modulating – agent is needed to optimize the BMP-2-induced generation of cartilaginous tissue from synovial explants.

It was the aim of the present study to ascertain whether the BMP-2-induced chondrogenesis of bovine synovial explants could be qualitatively influenced by the application of one of two different modulating chondrogenic agents, namely, TGF-ß1 or fibroblast growth factor 2 (FGF-2). Our data revealed that when BMP-2 was applied together with TGF-ß1, the synovial explants underwent a more thorough and homogenous differentiation into cartilaginous tissue. FGF-2 exerted no such effect. Furthermore, the downstream differentiation of the cells was arrested at an early stage of hypertrophy, and the hyaline-like properties of the neocartilage were improved.

## Materials and Methods

### Preparation and culturing of synovial explants

Synovial tissue was harvested from the metacarpal joints of 3- to 5-month-old bovine calves. The joints were purchased from a local butcher no later than 24 hours after the animals had been slaughtered. [For the use of such cadaver material, no approval by the cantonal authorities for animal experiments is required, since these are defined in the International Animal Welfare Act (Chapter 1, Article 3), as well as in the Swiss Animal Welfare Regulation (Article 3), as drawing on living organisms.] After washing, the material was cut into narrow tissue strips (3–5 mm in width×5–7 mm in length×1–2 mm in height) under sterile conditions. These explants were then sandwiched between two layers of agarose in 24-well tissue-culture plates, as previously described [27]. Each agarose-sandwiched explant was covered with 1 ml of serum-free medium [high-glucose Dulbecco's modified Eagle medium (Invitrogen, Scotland, UK) containing 1% ITS+™Premix (BD Biosciences, Bedford, MA), 1 mM proline (Sigma-Aldrich, St Louis, MO), ascorbic acid (50 µg/ml; Sigma-Aldrich) and gentamycin (50 µg/ml; Invitrogen). To induce chondrogenesis, the synovial explants were cultured for 4 (in selected cases for 6) weeks in the presence of BMP-2 [200 ng/ml; a generous gift from Pfizer (formerly Wyeth)]. FGF-2 (10 ng/ml; Calbiochem/Merck Biosciences, Darmstadt, Germany) or TGF-ß1 (10 ng/ml; Peprotech, Rocky Hill, NJ, USA) was introduced at the onset of the incubation and was present either during the first week of culturing alone or throughout the entire culturing period (4 or 6 weeks). In some instances, the synovial explants were exposed for 4 weeks to higher concentrations of BMP-2 (600 ng/ml or 2000 ng/ml). The growth factors were freshly re-introduced every 2 days when the medium was exchanged. The medium was supplemented with fresh ascorbic acid (25 µg/ml) on a daily basis. The synovial explants were cultured at 37°C in a humidified atmosphere containing 5% CO_2_.

### Histology and Histomorphometry

After culturing, the tissue explants were chemically fixed in phosphate-buffered saline (pH 7.4) containing 2% formaldehyde, and then embedded in paraffin. 5-µm-thick sections of the embedded material were prepared and stained with 1% Toluidine Blue (pH 2.5) for the histochemical detection of sulphated proteoglycans within a cartilaginous matrix (an indirect measure of chondrogenic differentiation). The sections were evaluated in a Nikon Eclipse E1000 light microscope using a DXM 1200F digital camera and Eclipsenet software (Nikon, Tokyo, Japan). The volume fraction of metachromasia in the tissue explants was assessed morphometrically, as previously described [27]. Individual cell (lacuna) volume was morphometrically estimated using the point-sampled intercept methodology [35] in conjunction with a systematic random-sampling protocol [36].

### Immunohistochemistry

Immunohistochemistry was performed on 5-µm-thick paraffin-embedded sections through the tissue explants, as previously described [27]. Murine monoclonal antibodies against aggrecan (clone HAG7D4, Serotec, Oxford, UK), type-II collagen (clone CII C1, Hybridoma Bank, Iowa City, IA) and type-IX collagen (clone D1–9, Hybridoma Bank), and lapine polyclonal antibodies against type-X collagen (ab58632, Abcam plc, Cambridge, UK) and COMP (a kind gift from Prof. D. Heinegård, Lund University, Lund, Sweden), were applied. Deparaffinized sections were exposed first to hyaluronidase [H3506 Sigma-Aldrich (8 mg/ml of sodium acetate buffer, pH 5.2)] and then to 3% skimmed bovine milk containing 1.5% equine or caprine serum to block non-specific antibody reactivity. Thereafter, they were incubated with one of the primary antibodies for 60 minutes at 37°C. A biotinylated form of an equine, anti-murine secondary antibody (Vector Laboratories, Burlingame, CA) was used to detect the antibodies against aggrecan, type-II collagen and type-IX collagen. A biotinylated form of a caprine, anti-lapine secondary antibody (Vector Laboratories) was applied to detect the antibodies against type-X collagen and COMP. Endogenous peroxidase activity was blocked with hydrogen peroxide. Immunoreactivity was enhanced by applying first the avidin-biotin-peroxidase complex (Vector Laboratories) and then biotinyl tyramide (Perkin Elmer, Waltham, MA, USA). Cell nuclei were counterstained with haematoxylin. The sections were evaluated and photographed in a Nikon Eclipse E1000 light microscope.

### Measurement of GAG-content

After culturing, the tissue explants were digested (overnight at 60°C) with 0.1% papain (Sigma-Aldrich) and 0.1% proteinase K (Sigma-Aldrich) in 1 mM CaCl_2_ containing 10 mM TRIS-HCl (pH 8) as a buffer [37]. The DNA-content of the digests was measured spectrofluorometrically using the High-Sensitivity Quant-iTT™ DNA Assay Kit (Molecular Probes, Eugene, OR). The GAG-content of the digests was determined colorimetrically after treatment with 1,9-Dimethylmethylene Blue (SERVA Electrophoresis, Heidelberg, Germany) [38]. Chondroitin sulphate (Sigma-Aldrich) was used as a standard. The weight of GAGs (in µg) was expressed per µg of DNA, as well as per mg of the initial wet weight of the explant.

### RNA extraction, reverse transcription and real-time PCR

After culturing, the tissue explants were stored at -70°C in RNAlater™ (Qiagen, Hilden, Germany). Prior to real-time PCR (Applied Biosystems 7900 instrument, Foster City, CA), the RNA was first isolated from samples that had been pulverized in a freezer-mill and then reverse transcribed using the Reverse Transcription System (Promega, Madison, WI), as previously described [27], [39]. An arbitrary calibrator was prepared from a mixture of the RNA that was isolated from the cartilage and the synovium of bovine metacarpal joints. Primers and probes were generated as previously described [30], [39]. Gene expression was normalized to the level of 18S rRNA using the formula 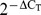, where C_T_ is the threshold cycle and ΔC_T_ = C_T_ (target gene)−C_T_ (18S rRNA). For each gene, the normalized level of mRNA in each sample was calculated relative to that in the calibrator (mixture of uncultured cartilage and synovium) using the formula 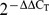, where ΔΔC_T_ = ΔC_T_ (sample)−ΔC_T_ (calibrator).

### Statistical analysis

Numerical data are represented as mean values together with the standard error of the mean (SEM). Comparisons between two sets of data were statistically appraised by an analysis of variances (ANOVA). Comparisons between multiple groups were evaluated by applying Dunnett's multiple comparison test. Statistical analyses were performed using SPSS software, version 11. 04 (SPSS Inc., Chicago, IL, USA). Statistical significance was set at a *p*-value of <0.05.

## Results

### Influence of FGF-2 on the BMP-2-induced chondrogenesis of synovial explants: histological, histochemical and biochemical evaluation

**Figure 1:**
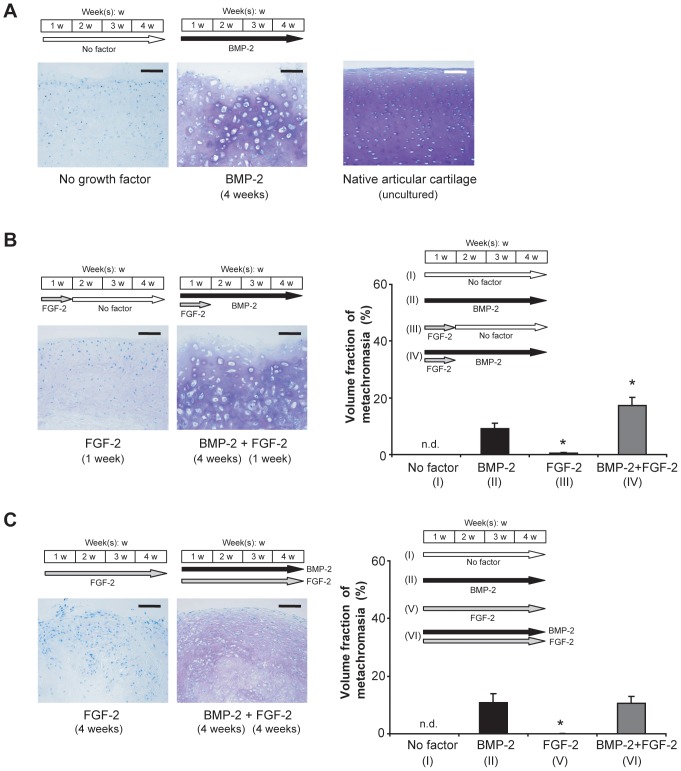
Influence of FGF-2 on the BMP-2-induced chondrogenesis of synovial explants (histochemical and histomorphometric analysis). A–C: Light micrographs of Toluidine-Blue-stained sections through bovine explants that had been cultured for 4 weeks under the indicated stimulation conditions, and graphs depicting the volume fraction of metachromasia in each case. In A, an image of metacarpal-joint cartilage is included as a positive control. BMP-2 was applied at a concentration of 200 ng/ml, and FGF-2 at one of 10 ng/ml. In the graphs, mean values for *n* = 4 animals (4 explants in each case) are represented together with the SEM. n.d.: not detectable; *: *p*<0.05 vs. “BMP-2” alone. Bars = 100 µm.

Synovial explants that had been cultured for 4 weeks in the absence of a growth factor evinced no signs of metachromasia after staining with Toluidine Blue (**Figure 1A**), and the morphology of the cells remained unchanged. Those that had been exposed to BMP-2 (200 ng/ml) for a like period (4 weeks) manifested marked metachromatic staining of the extracellular matrix and cell differentiation into chondrocytes, which were typically surrounded by pericellular lacunae (**Figures 1A** **,** **3C**). The boundary of each lacuna adumbrates the space originally occupied by the enclosed cell prior to its shrinkage during chemical processing [40]. The volume fraction of metachromasia varied between 10 and 20% of the total explant volume (**Figures 1B, 1C** **,** **3A, 3B**). The process of chondrocytic differentiation had progressed to a later stage of hypertrophy than is characteristic of native articular cartilage, as evidenced by the larger mean individual cell (lacuna) volume (**Figures 1A** **,** **3C**). However, in this *in-vitro* model, the extracellular matrix did not undergo calcification [as evidenced histochemically after staining with von Kossa's reagent [**41**]; data not shown].

**Figure 3:**
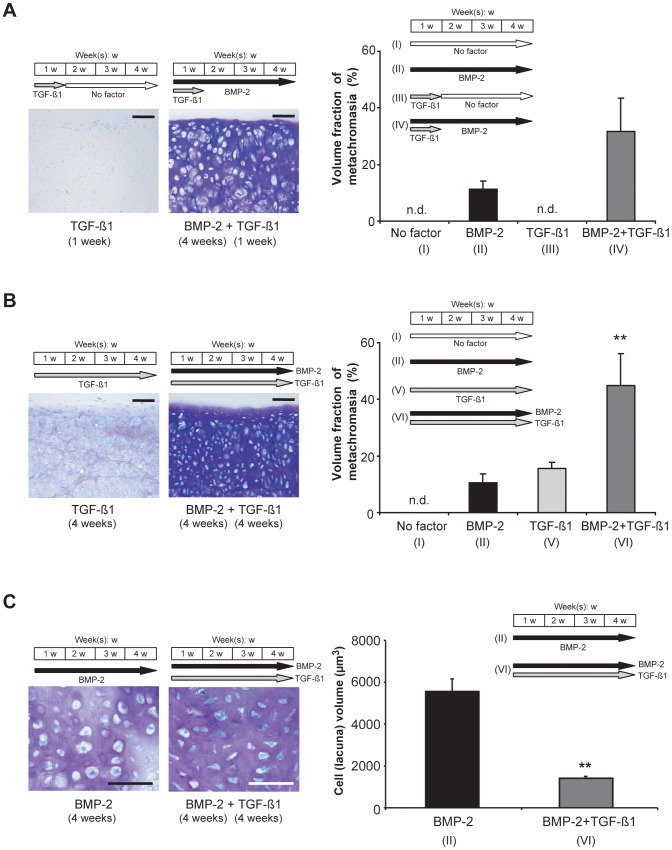
Influence of TGF-ß1 on the BMP-2-induced chondrogenesis of synovial explants (histochemical and histomorphometric analysis). A, B: Light micrographs of Toluidine-Blue-stained sections through bovine explants that had been cultured for 4 weeks under the indicated stimulation conditions (left), and graphs depicting the volume fraction of metachromasia in each case (right). BMP-2 was applied at a concentration of 200 ng/ml, and TGF-ß1 at one of 10 ng/ml. C: Light micrographs (left) of Toluidine-Blue-stained sections through bovine synovial explants that had been cultured for 4 weeks in the presence either of BMP-2 alone or of the TGF-ß1/BMP-2 combination, illustrating the size difference between the cell (lacuna) profiles in each case. The average cell (lacuna) volume was estimated histomorphometrically (right). Mean values for *n* = 4 animals (4 explants in each case) are represented together with the SEM. n.d.: not detectable; **: *p*<0.01 vs. “BMP-2”. Bars = 100 µm.

To assess the influence of FGF-2 on the BMP-2-induced chondrogenesis of synovial explants, this agent (10 ng/mL) was invariably introduced at the onset of incubation and was thereafter present either during the first week of culturing alone or throughout the entire 4-week culturing period. The effects of FGF-2 were tested both in the absence of BMP-2 and in its presence throughout the entire 4-week culturing period. In the absence of BMP-2, exposure of explants to FGF-2 during the first week of culturing alone led to weak metachromatic staining of the extracellular matrix but to no change in cell morphology (**Figure 1B**, left). The volume fraction of metachromasia was significantly lower than that achieved after exposure to BMP-2 alone (4 weeks) (**Figure 1B**, graph). In the presence of BMP-2 (4 weeks), exposure of explants to FGF-2 during the first week of culturing alone elicited a degree of metachromatic staining that was qualitatively similar to that achieved after exposure to BMP-2 alone (4 weeks); the attained stage of chondrocytic differentiation was likewise comparable [qualitatively similar cell (lacuna)-profile diameters] (**Figures 1A, 1B**). However, quantification of the volume fraction of metachromasia revealed this parameter to be 1.8-fold higher (*p*<0.05) after exposure to the FGF-2 (1^st^ week)/BMP-2 (4 weeks) combination than after stimulation with BMP-2 alone (**Figure 1B**, graph).

When explants were exposed to FGF-2 during the entire 4-week culturing period in the absence of BMP-2, metachromatic staining of the extracellular matrix was barely detectable and the cells evinced practically no signs of having been induced to undergo chondrogenesis (**Figure 1C**). In the presence of BMP-2 (4 weeks), exposure to FGF-2 during the entire 4-week culturing period evoked a degree of metachromasia which, although qualitatively less intense than that achieved after stimulation with BMP-2 alone (**Figures 1A, 1C**), was quantitatively (volume fraction) similar (**Figure 1C**, graph). Downstream chondrocytic differentiation had attained a less advanced stage than was apparent after stimulation with BMP-2 alone (late hypertrophy), as evidenced by the smaller cell (lacuna)-profile diameters (**Figures 1A, 1C**).

**Figure 2:**
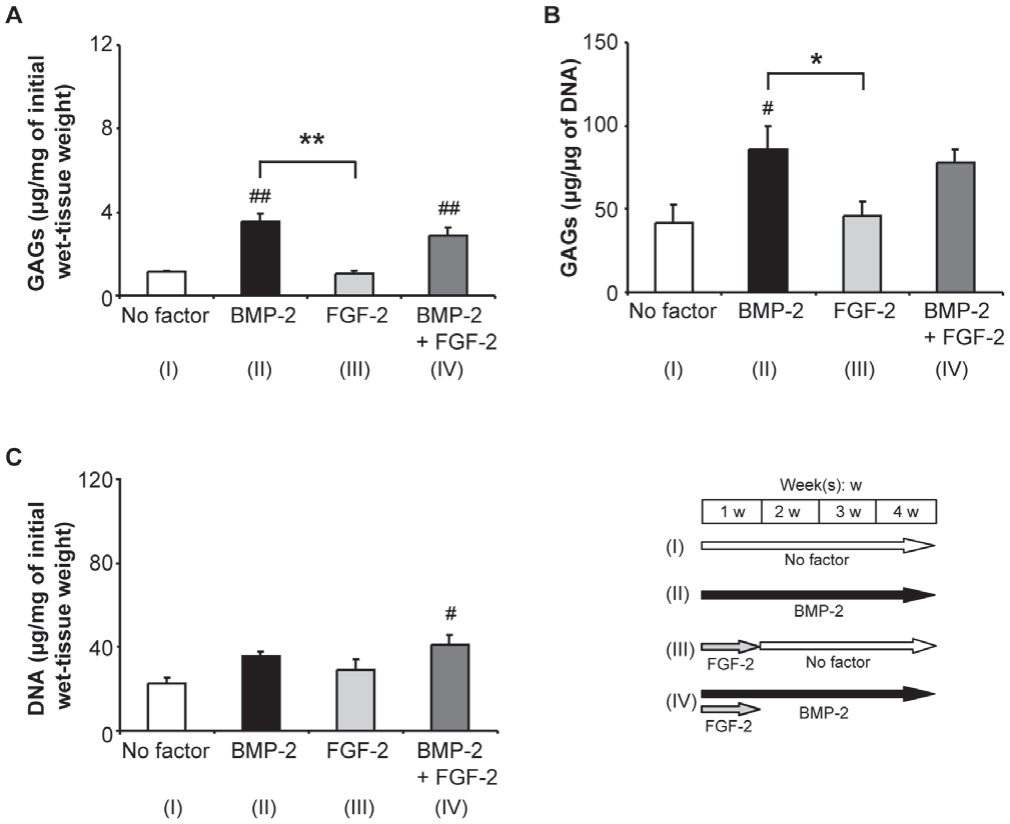
Influence of FGF-2 on the BMP-2-induced accumulation of GAGs in synovial explants (biochemical analysis). A, B: Graphs depicting the GAG-content of explants that had been cultured for 4 weeks under the indicated stimulation conditions, expressed either in µg per mg of the initial wet weight of the tissue sample (A) or in µg per µg of DNA (B). C: Graph depicting the DNA-content of explants that had been cultured for 4 weeks under the indicated stimulation conditions, expressed in µg per mg of the initial wet weight of the tissue sample. BMP-2 was applied at a concentration of 200 ng/ml, and FGF-2 at one of 10 ng/ml. Mean values for *n* = 4 animals (4 explants in each case) are represented together with the SEM. #: *p*<0.05 vs. “No factor”; ##: *p*<0.01 vs. “No factor”; *: *p*<0.05; **: *p*<0.01.

Since a limited, 1-week exposure to FGF-2 significantly enhanced the BMP-2-induced increase in the volume fraction of metachromasia, explants that had been so treated, as well as the corresponding positive and negative controls, were subjected to a quantitative biochemical analysis of their GAG-contents using the Dimethylmethylene-Blue assay. This parameter was expressed in µg per mg of the initial wet weight of the explant (**Figure 2A**) as well as in µg per µg of DNA (**Figure 2B**). After stimulation with BMP-2 alone (4 weeks), the GAG-content of the explants increased significantly relative to that of the negative control (no growth factor), irrespective of whether it was expressed per mg of the initial wet weight of the tissue or per µg of DNA. Exposure to FGF-2 alone (1^st^ week) elicited no increase in the GAG-content of the explants relative to that of the negative control, and when this agent was introduced (for 1 week) in the presence of BMP-2 (4 weeks), it did not potentiate the increase in this parameter that was achieved after stimulation with BMP-2 alone (**Figures 2A, 2B**). The DNA-content of the explants (expressed in µg per mg of the initial wet weight of the explant) increased significantly after exposure to the FGF-2 (1^st^ week)/BMP-2 (4 weeks) combination, but not after stimulation with either FGF-2 alone (1^st^ week) or BMP-2 alone (4 weeks) (**Figure 2C**).

### Influence of TGF-ß1 on the BMP-2-induced chondrogenesis of synovial explants: histological, histochemical and biochemical evaluation

To assess the influence of TGF-ß1 on the BMP-2-induced chondrogenesis of synovial explants, this agent (10 ng/ml) was invariably introduced at the onset of incubation and was thereafter present either during the first week of culturing alone or throughout the entire 4-week culturing period. The effects of TGF-ß1 were tested both in the absence of BMP-2 and in its presence throughout the entire 4-week culturing period. In the absence of BMP-2, exposure of explants to TGF-ß1 during the first week of culturing alone elicited no metachromatic staining of the extracellular matrix and no change in cell morphology (**Figure 3A**). In the presence of BMP-2 (4 weeks), exposure of explants to TGF-ß1 during the first week of culturing alone led to intense metachromatic staining of the extracellular matrix and to a differentiation of the cells into chondrocytes, which were typically surrounded by pericellular lacunae. Although the volume fraction of metachromasia tended to be higher than that achieved after stimulation with BMP-2 alone, the two mean values did not differ significantly from each other (**Figure 3A**, graph). The process of chondrocytic differentiation had progressed to the stage of late hypertrophy, as was the case after stimulation with BMP-2 alone (**Figures 1A** **,** **3A**).

When explants were exposed to TGF-ß1 during the entire 4-week culturing period in the absence of BMP-2, metachromatic staining of the extracellular matrix was very weak and the cells evinced practically no signs of having been induced to undergo chondrogenesis (**Figure 3B**, left). In the presence of BMP-2 (4 weeks), exposure of explants to TGF-ß1 during the entire 4-week culturing period led to very intense metachromatic staining of the extracellular matrix. The volume fraction of metachromasia was 4.8-fold higher than that achieved after stimulation with BMP-2 alone (**Figure 3B**, graph). The cells had differentiated into chondrocytes, which were typically surrounded by pericellular lacunae. However, the process of chondrocytic differentiation had been arrested at an earlier stage of hypertrophy than was apparent after stimulation with BMP-2 alone, as evidenced by the smaller cell (lacuna)-profile diameters (**Figures 1A** **,** **3B**). To confirm this qualitative impression, the volumes of the cells (lacunae) were morphometrically estimated according to stereological principles after culturing the explants in the presence of either BMP-2 alone (4 weeks) or the TGF-ß1 (4 weeks)/BMP-2 (4 weeks) combination. **Figure 3C** depicts, side by side, the typical appearance of explants that had been exposed to the two different protocols (left) and the results of the morphometric analysis of individual cell (lacuna) volume (right). The numerical data confirm the qualitative impression: the mean volume of the chondrocytes (lacunae) within explants that had been exposed to the TGF-ß1 (4 weeks)/BMP-2 (4 weeks) combination was smaller by a factor of 4 than those within explants that had been stimulated with BMP-2 alone.

**Figure 4:**
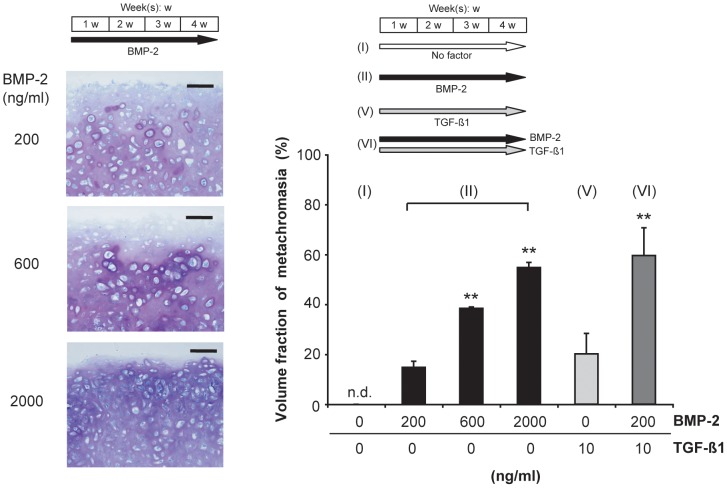
Dose-dependency of the BMP-2-induced chondrogenic differentiation of synovial explants. Light micrographs (left) of Toluidine-Blue-stained sections through explants that had been cultured for 4 weeks in the presence of BMP-2 at 200 ng/ml, 600 ng/ml or 2000 ng/ml, and graph (right) depicting the volume fraction of metachromasia in each case, as well as within explants that had been exposed for 4 weeks either to TGF-ß1 alone (10 ng/ml) or to the TGF-ß1 (10 ng/ml)/BMP-2 (200 ng/ml) combination. In the graph, mean values for *n* = 4 animals (4 explants in each case) are represented together with the SEM. n.d.: not detectable; **: *p*<0.01 vs. “BMP-2 (200 ng/ml)”. Bars = 100 µm.

We also investigated the influence of raising the concentration of BMP-2 when this agent was applied as the sole stimulant. Not only the intensity (**Figure 4**, left), but also the volume fraction of metachromasia increased in a dose-dependent manner, from 15% at 200 ng/ml, through 38% at 600 ng/ml, to 55% at 2000 ng/ml (**Figure 4**, graph). When explants were exposed to a combination of BMP-2 at 200 ng/ml (4 weeks) and TGF-ß1 at 10 ng/ml (4 weeks), the volume fraction of metachromasia (60%) was similar to that achieved after stimulation with BMP-2 alone (4 weeks) at a 10-fold higher concentration (2000 ng/ml). The volume fraction of metachromasia within explants that had been exposed to TGF-ß1 alone (10 ng/ml; 4 weeks) was comparable to that achieved after stimulation with BMP-2 alone (4 weeks) at the lowest concentration (200 ng/ml).

**Figure 5:**
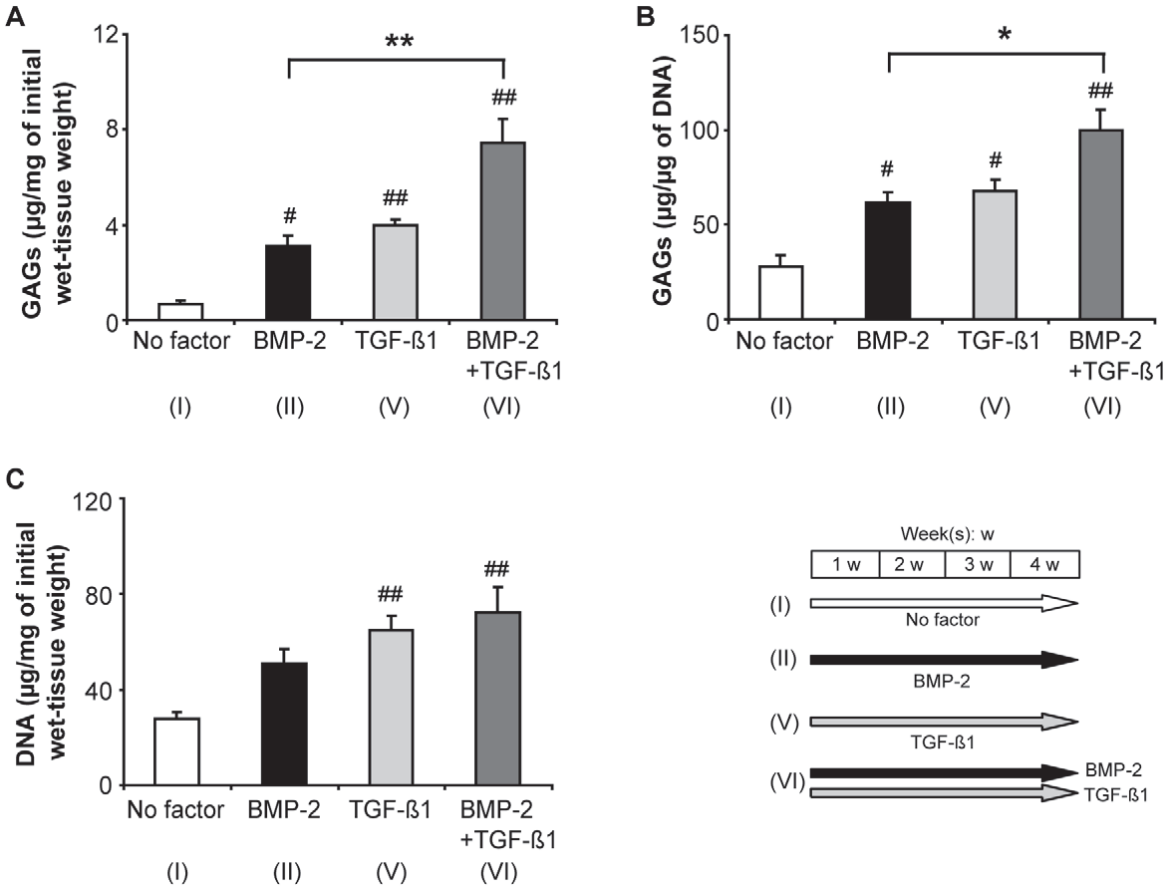
Influence of TGF-ß1 on the BMP-2-induced accumulation of GAGs in synovial explants (biochemical analysis). A, B: Graphs depicting the GAG-content of explants that had been cultured for 4 weeks under the indicated stimulation conditions, expressed either in µg per mg of the initial wet weight of the tissue sample (A) or in µg per µg of DNA (B). C: Graph depicting the DNA-content of explants that had been cultured for 4 weeks under the indicated conditions, expressed in µg per mg of the initial wet weight of the tissue sample. BMP-2 was applied at a concentration of 200 ng/ml, and TGF-ß1 at one of 10 ng/ml. Mean values for *n* = 4 animals (4 explants in each case) are represented together with the SEM. #: *p*<0.05 vs. “No factor”; ##: *p*<0.01 vs. “No factor”; *: *p*<0.05; **: *p*<0.01.

To confirm the potentiating effect of TGF-ß1 (10 ng/ml; 4 weeks) on the BMP-2-induced chondrogenesis of synovial explants at the lowest concentration of the latter agent (200 ng/ml; 4 weeks), we quantified the GAG-content of the tissue in the relevant groups (**Figure 5**). After stimulation with BMP-2 alone (200 ng/ml; 4 weeks), the GAG-content of the explants increased significantly relative to that in the negative control (no growth factor), irrespective of whether it was expressed per mg of the initial wet weight of the tissue or per µg of DNA (**Figures 5A, 5B**). Stimulation with TGF-ß1 alone (10 ng/ml; 4 weeks) led to a similar increase in the GAG-content of the explants, irrespective of whether it was expressed per mg of the initial wet weight of the tissue or per µg of DNA, there being no significant differences between the groups (*p*>0.05). However, after stimulation with the TGF-ß1 (10 ng/ml; 4 weeks)/BMP-2 (200 ng/ml; 4 weeks) combination, the GAG-content of the explants was significantly higher than after exposure to BMP-2 alone, irrespective of whether it was expressed per mg of the initial wet weight of the tissue (*p*<0.01; **Figure 5A**) or per µg of DNA (*p*<0.05; **Figure 5B**). The DNA-content of the explants increased after exposure to either TGF-ß1 alone or the TGF-ß1/BMP-2 combination, but not after stimulation with BMP-2 alone (**Figure 5C**).

### Influence of TGF-ß1 on the BMP-2-induced expression of cartilaginous genes within synovial explants

**Figure 6:**
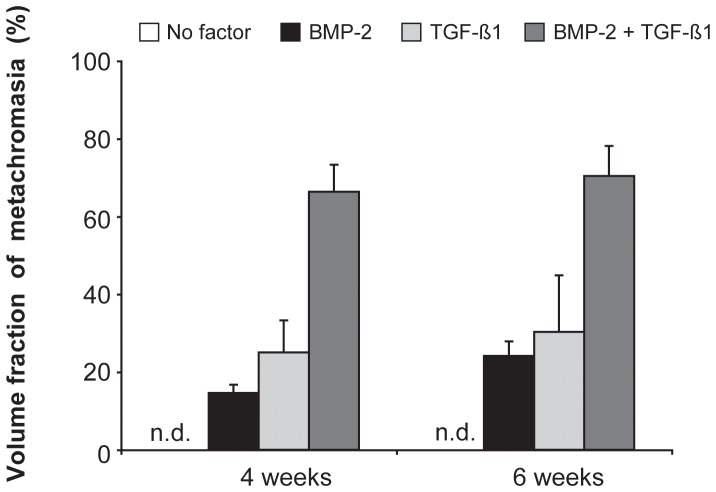
Influence of culturing time on the volume fraction of metachromasia in chondrogenically-stimulated synovial explants. Bovine explants were cultured for 4 or 6 weeks under the indicated stimulated conditions. BMP-2 was applied at a concentration of 200 ng/ml, and TGF-ß1 at one of 10 ng/ml. Each agent was introduced at the onset of the incubation and was present throughout its course. Mean values for *n* = 4 animals (4 explants in each case) are represented together with the SEM. n.d.: not detectable.

With a view to optimizing the stimulation conditions that are requisite for driving the chondrogenic differentiation of synovial explants into a hyaline type of articular cartilage, we monitored the gene-activity levels of a panel of selected markers after exposing the tissue to either BMP-2 alone (200 ng/ml), TGF-ß1 alone (10 ng/ml), or the TGF-ß1 (10 ng/ml)/BMP-2 (200 ng/ml) combination for 4 or 6 weeks. **Figure 6** depicts the volume fraction of metachromasia in each of the 3 groups at both time-points. Although this parameter tended to increase in a time-dependent manner in each group, the temporal differences did not attain statistical significance (*p*>0.05). The culturing period was extended from 4 to 6 weeks with a view to registering the gene activity for type-X collagen (a marker of chondrocytic hypertrophy [42]–[44]), which is raised to detectable levels only after the 4^th^ week of incubation [27]. In each group, the applied growth factors were present throughout the entire culturing period (4 or 6 weeks).

**Figure 7:**
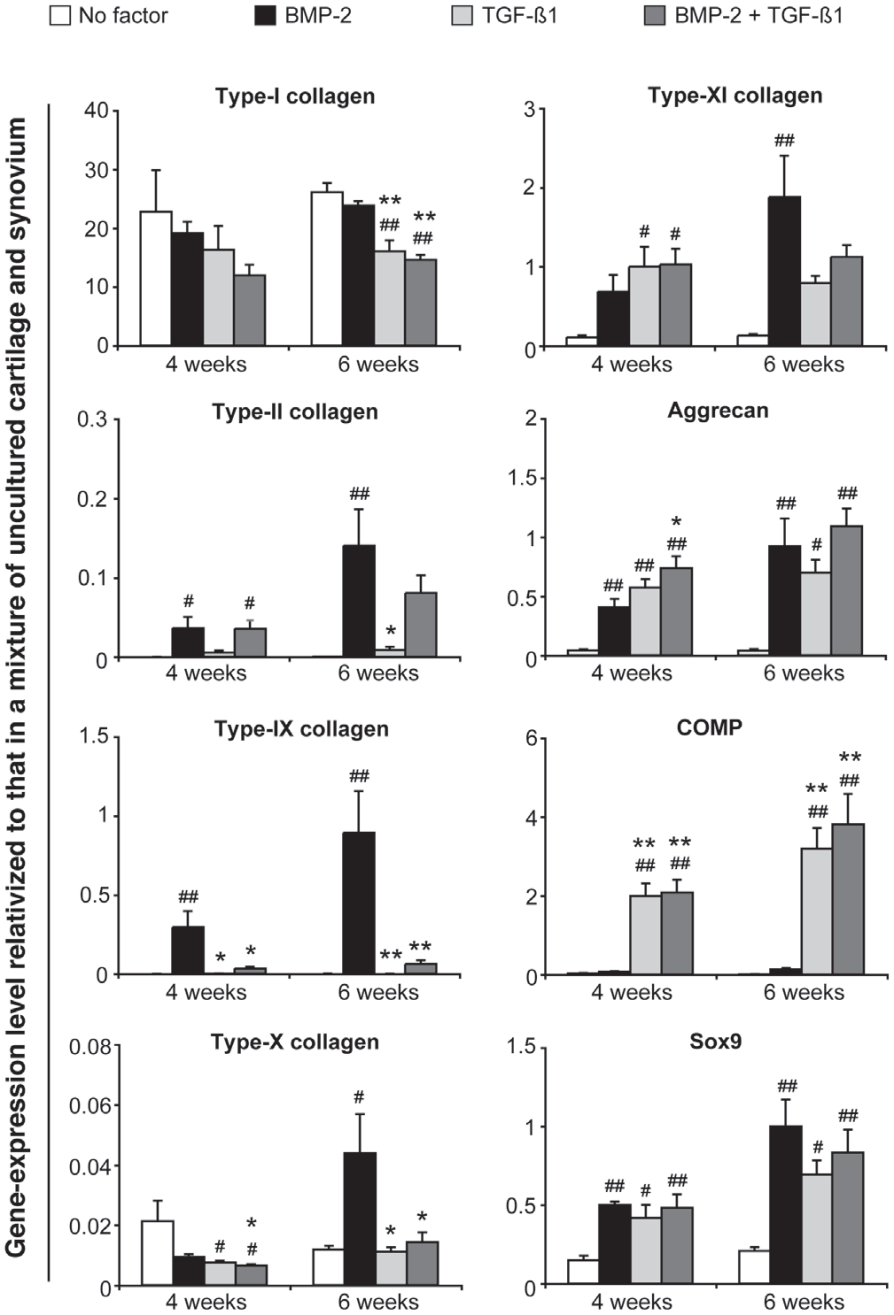
Influence of TGF-ß1 on the BMP-2-induced expression of cartilaginous genes in synovial explants. Bovine explants were cultured for 4 or 6 weeks under the indicated stimulation conditions. BMP-2 was applied at a concentration of 200 ng/ml, and TGF-ß1 at one of 10 ng/ml. Each agent was introduced at the onset of the incubation and was present throughout its course. The expression level of each gene (determined by a quantitative real-time PCR analysis) was related to that in a calibrator (a mixture of uncultured cartilage and synovium) after normalization to that of 18S rRNA. Mean values for *n* = 4 animals (4 explants in each case) are represented together with the SEM. #: *p*<0.05 vs. “No factor”; ##: *p*<0.01 vs. “No factor”; *: *p*<0.05 vs. “BMP-2”; **:*p*<0.01 vs. “BMP-2”.

After stimulation with BMP-2 alone for 4 or 6 weeks, the gene-expression levels of type-II collagen, type-IX collagen, aggrecan and Sox9 increased significantly in a time-dependent manner relative to those in the control group (no growth factor) (**Figure 7**). The gene-expression levels of collagen types X and XI increased significantly only between the 4^th^ and the 6^th^ weeks. After stimulation with TGF-ß1 alone for 4 weeks, a different pattern of gene activity was revealed: the levels of type-XI collagen, aggrecan, COMP and Sox9 increased significantly, whereas those of type-X collagen decreased. An extension of the culturing period from 4 to 6 weeks led to further increases in the gene-expression levels of aggrecan, COMP and Sox9, and to a significant decrease in that of type-I collagen. Hence, in comparison with the situation pertaining after exposure to BMP-2 alone, stimulation with TGF-ß1 alone led, after 4 weeks, to an enhancement in the gene-expression level of COMP, but to a lowering in that of type-IX collagen, and, after 6 weeks, likewise to an enhancement in the gene-expression level of COMP, but to a lowering in that of each collagen type except type XI. After stimulation with the TGF-ß1/BMP-2 combination for 4 weeks, the gene-expression levels of aggrecan and COMP were significantly higher, whereas those of collagen types IX and X were significantly lower, than after stimulation with BMP-2 alone. When the culturing period was extended from 4 to 6 weeks, the gene-expression level of COMP was higher, whereas those of collagen types I, IX and X were lower, than after stimulation with BMP-2 alone.

### Influence of TGF-ß1 on the BMP-2-induced deposition of cartilaginous proteins within synovial explants

**Figure 8:**
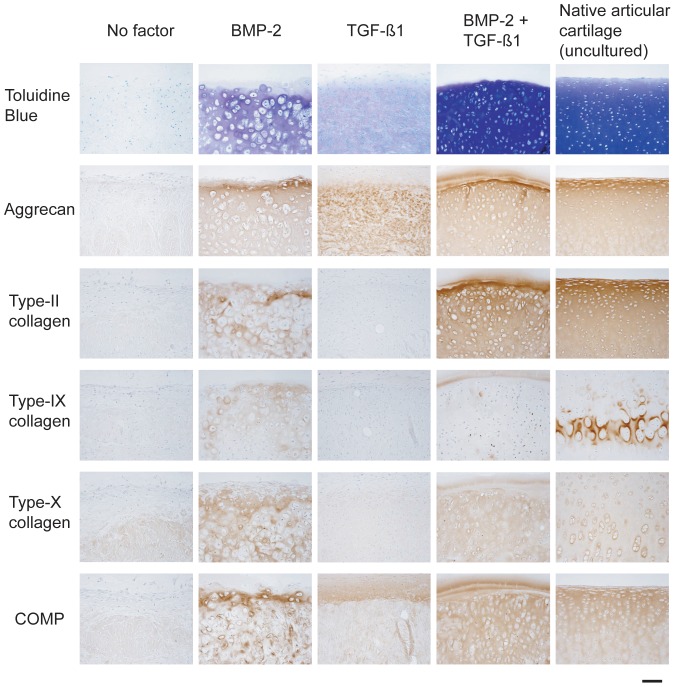
Influence of TGF-ß1 on the BMP-2-induced deposition of cartilaginous proteins in synovial explants (immunohistochemical analysis). Light micrographs of sections through bovine explants that had been cultured for 6 weeks under the indicated stimulation conditions and then either stained with Toluidine Blue (1^st^ row), or treated for the immunohistochemical detection of aggrecan (2^nd^ row), type-II collagen (3^rd^ row), type-IX collagen (4^th^ row), type-X collagen (5^th^ row) or COMP (6^th^ row). BMP-2 was applied at a concentration of 200 ng/ml, and TGF-ß1 at one of 10 ng/ml. Each agent was introduced at the onset of the 6-week culturing period and was present throughout its course. Native (uncultured) metacarpal-joint cartilage (final vertical column) served as a positive control. Cell nuclei (2^nd^ to 6^th^ rows) were counterstained with haematoxylin. All photomicrographs are represented at the same final magnification. Bar = 100 µm.

An immunohistochemical analysis was conducted to evaluate the modulatory effects of TGF-ß1 on the BMP-2-induced post-translational production and secretion of key cartilaginous proteins, namely, aggrecan, collagen types II, IX and X, and COMP (**Figure 8**). Synovial explants were cultured for 6 weeks in the absence of a growth factor (negative control), or in the presence of either BMP-2 alone (200 ng/ml), TGF-ß1 alone (10 ng/ml), or the TGF-ß1 (10 ng/ml)/BMP-2 (200 ng/ml) combination. In each group, the applied growth factors were present throughout the entire 6-week culturing period. Bovine articular cartilage that had been derived from the metacarpal joint served as a positive control. Within the extracellular matrix of native articular cartilage, immunoreactivity for aggrecan and type-II collagen, as well as that for COMP, was observed from the superficial zone down to the lower radial one (**Figure 8**). Immunostaining for type-IX collagen occurred predominantly in the zone of calcified cartilage, and was more intense in this region than in any other. Immunoreactivity for type-X collagen was inhomogeneously distributed throughout the extracellular matrix in the transitional and the upper radial zones (small cells) (not illustrated in Figure 8), but was strongest within the pericellular compartment in the lower radial one (hypertrophic chondrocytes) (**Figure 8**).

Synovial explants that had been cultured in the absence of a growth factor (negative control) revealed no detectable signs of immunoreactivity for either type-II or type-IX collagen; weak signals were registered for aggrecan, type-X collagen and COMP. Explants that had been stimulated with BMP-2 alone registered positive for each of the tested proteins. After stimulation with TGF-ß1 alone, immunostaining of the tissue samples was observed only for aggrecan, not for any of the tested collagen types (II, IX and X). Immunoreactivity for COMP was confined to the upper layer of agarose within which the explants were embedded (see Materials and Methods), and into which some of the peripheral synovial cells had migrated. Compared to explants that had been stimulated with BMP-2 alone, those that had been exposed to the TGF-ß1/BMP-2 combination manifested more homogeneously-distributed immunoreactivity for aggrecan, type-II collagen, type-X collagen and COMP; that for type-IX collagen was appreciably reduced; it was confined to the upper layer of agarose within which the explants were embedded and to the pericellular matrix around scattered cells throughout the depth of the tissue samples. It is noteworthy that in native articular cartilage, immunoreactivity for type-X collagen occurred predominantly within the pericellular matrix that surrounded hypertrophic chondrocytes, whereas in the explants that had been exposed to the TGF-ß1/BMP-2 combination, it was more homogeneously distributed throughout the extracellular matrix as a whole and was not confined to any particular stratum. Although immunoreactivity for type-X collagen was more homogeneously distributed after exposure to the TGF-ß1/BMP-2 combination than after stimulation with BMP-2 alone, the overall intensity of staining was weaker.

## Discussion

FGF-2 is described in the literature to be a highly potent mitogen for various cell-types of mesodermal and neuroectodermal origin [45]. For this reason, it is often used to stimulate the expansion of MSCs *in vitro* [46], [47]. Human bone-marrow-derived MSCs that have been expanded in the presence of FGF-2 not only proliferate more rapidly but also manifest a higher chondrogenic potential than those grown in its absence [48]–[51]. Similar effects have been observed for MSCs derived from adipose or synovial tissue [52], [53]. However, if FGF-2 is introduced into the growth medium at a juncture corresponding to the chondrogenic-differentiation phase of the MSCs, the reported effects of the agent are conflicting. On the one hand, FGF-2 has been reported to enhance the TGF-ß1-induced chondrogenesis of lapine periosteal explants [54] and of adipose-tissue-derived murine MSCs [52], whilst on the other, it has been described to suppress the TGF-ß1-induced – and to completely abolish the potentiating effect of BMP-6 on the TGF-ß3-induced – chondrogenesis of adipose-tissue-derived human MSCs [55], [56].

In the present study, FGF-2 was applied in combination with BMP-2 to cultures of bovine synovial explants. When FGF-2 was applied during the first week of culturing alone, it enhanced the BMP-2-induced increase in the volume fraction of metachromasia within the explants, whereas when it was present throughout the entire 4-week culturing period it did not. We do not yet understand why the potentiating effect of FGF-2 on the BMP-2-induced chondrogenesis of synovial explants depends upon the duration of exposure to the agent. The potential of FGF-2 to induce variable and even opposing effects *in vivo* as well as *in vitro* is, however, well known. FGFs are essential for skeletal development, and during this process, their influence has been described to be both stage- and region-specific [57], [58]. Likewise *in vitro*, FGFs have been shown to regulate the osteogenic differentiation of neonatal calvarial osteoblastic cells as well as the chondrogenesis of bone-marrow-derived MSCs in a stage-specific manner [59], [60]. These findings suggest that the expression of FGF-receptors on osteo- or chondroprogenitor cells is confined to a narrow temporal window during the process of their differentiation. In our experimental set-up, this window fell within – and had been passed through by the end of – the first week of stimulation with FGF-2.

Although FGF-2 enhanced the BMP-2-induced increase in the volume fraction of metachromasia within the explants, its influence was not sufficiently potent to augment the GAG-content of the tissue samples. TGF-ß1, on the other hand, enhanced not only the BMP-2-induced increase in the volume fraction of metachromasia within the explants (in a manner that was not confined to a temporal window), but also the GAG-content of the tissue samples. The potentiating effect of TGF-ß1 on the BMP-2-induced production of GAGs (aggrecan) was confirmed also at the gene- (**Figure 7**) and protein- (**Figure 8**) expression levels. On the other hand, TGF-ß1 enhanced the BMP-2-induced increase in the post-translational deposition of type-II collagen without influencing its gene-expression level. Although we cannot as yet account for this phenomenon, we suspect that the discrepancy may arise from a possible interaction between the type-II-collagen protein and other components of the cartilaginous matrix. COMP, for example, is known to bind to collagenous proteins [61]–[63] and to enhance their fibrillogenesis [64]. In the present study, TGF-ß1 enhanced the BMP-2-induced increase in both the gene- and the protein-expression levels of COMP. This being the case, it is conceivable that the type-II-collagen protein – stabilized by COMP – was retained within, rather than leaching out of, the cartilaginous matrix, thereby resulting in an immunohistochemical signal for the protein itself that was disproportionately high relative to the expression level of its gene.

In combination with BMP-2, TGF-ß1 was capable of arresting, at an early juncture, the downstream hypertrophic differentiation of chondrocytes, which, after stimulation with the former agent alone, progresses to the terminal stage that is inextricably associated with matrix mineralization. This anti-hypertrophic effect of TGF-ß1 on the BMP-2-induced chondrogenesis of bovine synovial explants was discovered by ourselves several years ago, and although the finding was not documented at the time, it was communicated in an oral presentation at the 7^th^ World Congress of the International Cartilage Repair Society (2007). Subsequently, the effect was confirmed using pellet-cultures of isolated human synovial MSCs and BMP-7 instead of BMP-2 as a stimulant [65]. The histomorphological findings presented in this publication were, however, of a descriptive nature only; they were not substantiated either by histomorphometry or by an analysis of the gene- and protein-expression levels. In the present study, these analyses were undertaken. TGF-ß1 was found to suppress the BMP-2-induced increases in both the gene- (marked effect at 6 weeks) and the protein- (appreciable effect at 6 weeks) expression levels of type-X collagen, which is a marker of chondrocytic hypertrophy.

In growth-plate cartilage, the type-X-collagen protein is expressed predominantly by hypertrophic chondrocytes [42]–[44], and its well-known calcium-binding properties appear to play a role in the mineralization process that they initiate at a late stage of differentiation [66]. In articular cartilage, on the other hand, the type-X-collagen protein is expressed not only in the zone of calcification, but also in other regions of the tissue layer [67], [68]. In the present study, the immunohistochemical analysis of bovine metacarpal-joint cartilage revealed the type-X-collagen protein to be expressed throughout the extracellular matrix in the transitional and the upper radial zones (small cells), but almost exclusively within the pericellular compartment in the lower radial one (hypertrophic chondrocytes) (**Figure 8**). Further investigations will be necessary to elucidate the biological role of the type-X-collagen protein in the zones containing the small (non-hypertrophic) chondrocytes.

In native bovine articular cartilage, the type-IX-collagen protein was expressed exclusively in the zone of calcification, which accords with the findings in a porcine model [69], although in foetal avian sternal cartilage, it is distributed throughout the entire tissue layer [70]. The role played by type-IX collagen in the zone of calcification has not been clearly defined. In foetal avian cartilage, it has been shown to stabilize the cross-linkage of collagen fibrils [70]. In transgenic mice that lack the type-IX-collagen gene, skeletal morphogenesis follows a normal course up to the time of parturition. However, thereafter, when the joints of the animals have to bear weights, the articular-cartilage layer undergoes progressive degeneration. This latter finding indicates that type-IX collagen plays an important role in stabilizing the mechanical properties of the articular-cartilage layer in weight-bearing regions [71], [72]. In the present study, TGF-ß1 suppressed the BMP-2-induced increases in both the gene- and the protein-expression levels of type-IX collagen. Whether this effect is reflected in a measurable influence on the mechanical stability of the explants is not yet known, but the issue could be clarified by subjecting the tissue samples to appropriate biomechanical tests (currently under investigation).

With a view to the engineering of articular cartilage in the context of clinical repair, the finding that TGF-ß1 is capable of arresting the BMP-2-induced downstream differentiation of synovial MSCs into chondrocytes at an early stage of hypertrophy represents an important step towards the generation of a hyaline – rather than the usual fibrous [73] – type of repair tissue. *In vivo*, the complete downstream differentiation of cells into terminal hypertrophic chondrocytes is inextricably coupled with mineralization of the extracellular matrix. The potency of BMP-2 in stimulating the chondrogenesis of MSCs is advantageous on the one hand in that it assures an efficacious differentiation response, but disadvantageous on the other in so far as the process is catapulted rather too efficaciously towards completion in the downstream direction, resulting in what is for articular cartilage a hypertrophic overshoot but for the growth plate a necessity. In epiphyseal-plate cartilage, the mean volume of individual terminal hypertrophic chondrocytes varies between 5,000 and 20,000 µm^3^, according to the phase of growth [31], [32]. In articular cartilage, the mean volume of individual hypertrophic chondrocytes in the lower radial zone (which abuts on the calcified region) ranges from 2,000 to 4,000 µm^3^ [33], [34], with little variation between species [74]. In the present study, the mean volume of individual chondrocytes in the explants was 5,600 µm^3^ after stimulation with BMP-2 alone and 1,400 µm^3^ after exposure to the TGF-ß1/BMP-2 combination (**Figure 3**), which approximates to the terminal size achieved in the lower radial zone of native articular cartilage [33], [34].

The concomitant application of diverse TGF-ß isoforms and BMPs has been demonstrated also by other investigators to potentiate the chondrogenic effects of either agent alone on MSCs. For example, using bone-marrow-derived human MSCs, BMP-2, BMP-6 and BMP-7 have been individually shown to enhance TGF-ß3-induced chondrogenesis, evidence for which was furnished by an up-regulation of the genes for aggrecan and type-II collagen [75], by an increase in the total weight of the pellets and in their metachromatic staining for GAGs [76], and by the deposition of augmented quantities of proteoglycans, type-II collagen and Sox9 [77]. And also using human MSCs of adipose-tissue origin, a combination of BMP-7 and TGF-ß2 has been described to enhance the quantity of GAGs and the expression of the type-II-collagen gene over and above the levels that were achieved after stimulation with either of the factors alone [78]. However, in these earlier reports, no mention was made of an anti-hypertrophic effect of the applied TGF-ß on BMP-induced chondrogenesis. The absence of the effect in these former studies could be attributable to several factors. Firstly, the anti-hypertrophic effect of TGF-ßs may depend upon the tissue source of the MSCs. The responsivity of MSCs to a given growth factor has indeed been shown to vary according to their tissue origin [30]. Although some of the combined effects of BMP-2 and TGF-ß on the chondrogenesis of isolated, aggregate-cultured human MSCs of synovial origin have been described [79], [80], an anti-hypertrophic influence of the latter agent was not apparent, since the applied concentration of BMP-2 was too low to induce the full downstream differentiation of the cells into terminal chondrocytes [79]. The effects of stimulating with BMP-2 alone were not documented [80]. Secondly, the anti-hypertrophic effect of TGF-ßs on MSCs might depend upon the presence of their natural extracellular matrix. Isolated MSCs are stripped of the niche information to which they are subject *in situ*, and their extrication from it can have a marked influence on their reactivity [30]. Thirdly, the anti-hypertrophic effect exerted by TGF-ßs may depend upon the nature of the applied isoform, or, fourthly, upon the concentration at which it is used [81].

Indirect evidence of the anti-hypertrophic effect of TGF-ß1 is, however, available for other cell systems. For example, using isolated, aggregate-cultured rat periosteal cells, TGF-ß1 has been shown to inhibit the BMP-2-induced expression of the genes for type-X collagen and osteocalcin [82]. Similarly, TGF-ß1 has been reported to suppress the terminal differentiation of both lapine [83] and rat [84] growth-plate chondrocytes *in vitro*. And in living mice, deletion of the gene for smad3, which is one of the intracellular mediators of TGF-induced signalling cascades, led to an enhancement of the terminal differentiation of articular chondrocytes [85]. This latter finding corroborates those of the present study. TGF-ß-induced signalling would thus appear to play a crucial role in regulating the hypertrophy of chondrocytes. However, whether this effect is exerted in humans and other primates, as well as in non-primates, remains to be clarified.

In summary, although BMP-2 is capable of inducing the chondrogenesis of bovine synovial explants, the cartilaginous tissue formed is neither homogeneously distributed nor of an exclusively hyaline type, and the process of downstream differentiation progresses all the way to the stage of terminal hypertrophy (as occurs physiologically in growth-plate and foetal – but not in articular – cartilage). TGF-ß1 enhances the BMP-2-induced chondrogenic differentiation of synovial explants, improves the hyaline-like properties of the cartilaginous tissue formed, and arrests the process of hypertrophy at an early stage, which corresponds to that attained in the lower radial zone of native articular cartilage.
